# The Effects of Walking Exercise Using Water Inertial Load on Dynamic Balance Ability and Pain in Women Aged 65 Years and Older with Knee Osteoarthritis: A Randomized Clinical Trial

**DOI:** 10.3390/jfmk10040469

**Published:** 2025-12-03

**Authors:** Moon Jung Chu, Chae Kwan Lee, Hyun Ju Kim, Il Bong Park

**Affiliations:** Department of Sports Medicine, Busan University of Foreign Studies, Busan 46234, Republic of Korea; 20255416@office.bufs.ac.kr (M.J.C.); 20246001@bufs.ac.kr (C.K.L.); 20246204@bufs.ac.kr (H.J.K.)

**Keywords:** inertial water load, Aqua Vest, Y-Balance Test, center of pressure, neuromuscular control, postural control, proprioception

## Abstract

**Background**: Aging reduces proprioception and neuromuscular control, leading to impaired balance and increased pain, particularly in women aged 65 years and older with knee osteoarthritis (KOA). Therefore, this study investigated the effects of walking exercise using a water inertial load on dynamic balance and pain in this population. **Methods**: Thirty-four women aged 65 years and older with KOA were recruited and randomly assigned to an experimental group (Aqua Vest group, *n* = 17) or a control group (weighted vest group, *n* = 17). After dropout, data from 28 participants were included in the analysis. Both groups performed the same walking exercise program twice weekly for eight weeks. Dynamic balance was evaluated using the Y-Balance Test (YBT), postural control using center of pressure (COP), and pain using the visual analogue scale (VAS). Data were analyzed using an analysis of covariance (ANCOVA) for between-group comparisons and the Wilcoxon signed-rank test for within-group changes. **Results**: The experimental group showed significantly greater improvement in YBT composite scores compared with the control group (*p* < 0.05). COP velocity showed a significant improvement in the anterior direction (*p* < 0.05), whereas no significant differences were observed in other directions. Pain decreased in both groups, but no significant between-group difference was observed. **Conclusions**: Eight weeks of walking exercise using a water inertial load significantly improved dynamic balance and showed a positive trend toward pain reduction in women aged 65 years and older with KOA. These findings suggest that the nonlinear inertial characteristics of water may enhance sensory–motor integration and postural control, contributing to improved balance performance.

## 1. Introduction

Accelerated biological aging in adults is associated with an increased prevalence of osteoarthritis (OA) [1], a trend that is particularly pronounced among women and older adults aged 65 years and older. Moreover, aging acts as a major risk factor for the incidence of KOA [2]. KOA is characterized by pain, stiffness, and functional limitations, resulting in multi-tissue structural alterations across cartilage, bone, synovium, and other intra-articular components [3]. These structural changes lead to pain and functional decline, acting as key contributors to the overall reduction in daily performance ability and quality of life among women aged 65 years and older [4].

Women are known to be more susceptible to knee joint disorders due to anatomical structure and physiological factors [5]. Landry et al. [6] reported that, in situations requiring unexpected lateral movements, women showed differences in activation of the rectus femoris and gastrocnemius muscles, as well as imbalances between medial and lateral musculature compared to men, which is associated with an increased risk of knee injury. In addition, a rapid decline in estrogen levels after menopause leads to reduced muscle mass [7]. Women with lower lower-limb muscle mass exhibit greater KOA severity, suggesting that muscle loss may be associated with deteriorating knee joint health [8]. Furthermore, women with higher body mass index (BMI) show a significantly increased risk of developing OA, and this trend has been consistently reported regardless of diagnostic criteria [9].

Pain in OA is not limited to cartilage damage alone but originates from complex structural damage and inflammatory responses, including subchondral bone alterations, bone marrow lesions, and synovitis [10]. Additionally, approximately 20–40% of patients with knee OA experience neuropathic-like pain, indicating that neural mechanisms beyond structural pain should also be considered [11]. As a result, pain and functional limitations associated with KOA contribute to disability and overall decline in quality of life [12].

Moreover, proprioceptive deficits have been reported in patients with KOA, and the effects of proprioceptive-based exercise aimed at improving this have been demonstrated [13]. The ability to maintain balance in daily life is essential [14], and, in particular, dynamic balance refers to the ability to maintain a stable posture during movement and to quickly recover balance when it is disrupted [15]. In older adults aged 65 years and older, physiological and biomechanical changes accumulate over time, leading to gradual reductions in postural stability, with a particularly marked decline after the age of 70 [16]. In addition, the ability to restore the body’s center of mass in unstable environments or in response to unexpected external perturbations worsens [17], and older adults aged 65 years and older demonstrate significantly reduced ability to control dynamic stability compared to younger adults [18].

These changes may lead to more cautious movement and limit adaptability to novel environments. Therefore, accurate assessment of dynamic balance is essential, and YBT has been reported as a reliable and valid tool for objectively evaluating balance function [19,20]. Shaffer et al. [21] reported high test–retest reliability for YBT across assessors, regardless of their level of experience. In addition, recent research in older adults has confirmed that YBT is a valid tool that sensitively reflects age-related declines in postural control [22]. However, the conventional YBT primarily focuses on reach distance as an index for functional range of motion and lower-limb strength asymmetry, and it has been judged to have limitations in sufficiently reflecting the quality of postural control.

To address this limitation, the present study incorporated COP as an additional parameter to quantitatively evaluate the postural stabilization process during YBT performance. In particular, COP velocity sensitively reflects the ability to regulate body weight shifts during movement and is a useful variable for quantifying postural stabilization strategies in dynamic balance tasks [23,24].

Accordingly, by conducting a concurrent analysis of the spatiotemporal COP parameters that could not be captured by the conventional YBT, this study holds significance in its comprehensive assessment of the qualitative aspects of motor control.

Walking exercise is known to be the most common form of physical activity among adults aged 65 years and older [25]. However, a randomized controlled trial involving patients with severe KOA reported that 70 min per week of moderate-intensity walking improved cardiovascular health but did not result in significant reductions in knee pain [26]. This suggests that walking alone has limitations in alleviating pain, highlighting the need for new interventions that can simultaneously improve both dynamic balance and pain. Aqua Vest, a recently spotlighted exercise device, is partially filled with water, allowing the internal water to move freely during movement and generate unpredictable, multi-directional perturbations. This inertial load of water continuously challenges the body’s postural stability, and through repeated responses to this instability, proprioception and neuromuscular control are enhanced [27,28,29]. Unstable environment training utilizing water inertial load has been shown to improve balance ability and dynamic stability, with one study reporting improved reach distances in all three YBT directions anterior (ANT), posteromedial (PM), and posterolateral (PL) [28]. Furthermore, water-based inertial load exercise was found to enhance postural stability during stair ambulation and reduce pain in women with knee OA [27]. Notably, Kim and Park [29] reported that dynamic stability training using water inertial load improved coordination control of lower-limb joints and postural stability through repeated perturbations, suggesting that the instability caused by water movement promotes sensory–motor responses and contributes to improved balance control. Meanwhile, although walking is the most common form of physical activity among older adults [25], existing studies have primarily focused on cardiovascular benefits and pain relief [26], with few reporting quantitative analyses of dynamic balance. Thus, simple walking exercise alone may not be sufficient to fully explain improvements in dynamic balance regulation.

Therefore, the purpose of this study was to investigate the effects of walking exercise with inertial load of water (Aqua Vest walking exercise) on dynamic balance and pain in women aged 65 years and older with KOA. Additionally, by conducting COP analysis during YBT performance, the study aimed to quantitatively evaluate the qualitative aspects of motor control and explore changes in postural stabilization strategies that could not be identified through conventional walking exercise.

## 2. Materials and Methods

### 2.1. Participants

This study included women aged 65 years and older residing in region B who had been diagnosed with KOA by a physician and were recommended to receive exercise therapy. Prior to participation, all individuals received a thorough explanation of the study’s purpose and procedures, and written informed consent was obtained. The study was conducted in accordance with the Declaration of Helsinki and was approved by the Joint Institutional Bioethics Committee (IRB approval number: P01-202509-01-045). In addition, the study was registered in the Clinical Research Information Service (CRIS), and the clinical trial registration number is KCT0011140.

Participants were recruited through B University and a local community welfare center. A total of 34 participants were enrolled and randomly assigned to either the experimental group (aqua-vest training group, *n* = 17) or the control group (weighted vest training group, *n* = 17) using a computer-generated block randomization method (block size = 4) by an independent researcher. Group allocation was concealed in sealed opaque envelopes until the start of the intervention.

The required sample size was calculated using G*Power 3.1 software by referring to a previous study [29]. Based on an effect size of 0.25, a significance level of α = 0.05, and a statistical power of 1 − β = 0.80, a total of 28 participants (14 in each group) were required. Considering a dropout rate of approximately 20%, the final sample size was set at 34.

However, six participants withdrew due to personal health-related reasons unrelated to the intervention; therefore, data from 28 participants were included in the final analysis.

Specifically, two participants withdrew due to acute infectious diseases (e.g., influenza), two due to incidental injuries related to daily activities (e.g., fractures caused by falls), and two due to non-exercise-related internal medical conditions requiring treatment.

Importantly, no adverse events or exercise-related complications were reported.

Although some participants withdrew, the final sample size (*n* = 28) met the minimum sample size estimated by the a priori power analysis; therefore, statistical power was considered to have been maintained at a sufficient level.

The criteria for participant selection are as follows:

Inclusion Criteria:

(1) Individuals diagnosed with KOA with Kellgren–Lawrence (K-L) Grade 2–3 according to radiological assessments
(2) Individuals who have been diagnosed with KOA for more than 3 years
(3) Individuals without congenital deformities or musculoskeletal disorders in the foot, pelvis, or spine

Exclusion Criteria:

(1) Individuals with K-L Grade 0–1 or Grade 4
(2) Individuals who have undergone lower limb surgery within the past year
(3) Individuals with moderate or severe cardiovascular or respiratory diseases
(4) Individuals with uncontrolled hypertension or diabetes
(5) Individuals who have engaged in regular exercise during the past 6 months
(6) Individuals with neurological disorders or acute inflammation and joint swelling
(7) Individuals taking medications that could affect pain perception or balance
(8) Individuals diagnosed with sarcopenia or other muscle-wasting disorders

**Figure 1:**
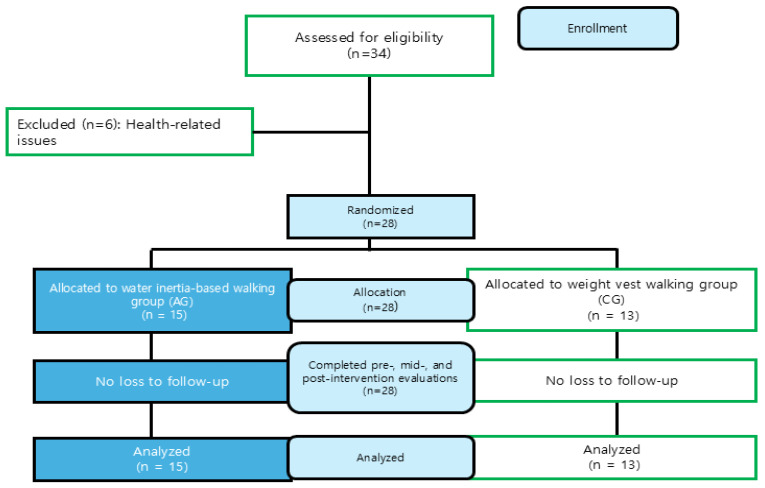
Flow diagram of the study participants.

Statistical analyses verified baseline equivalence between the two groups, with no significant differences in height (t = −0.109), weight (t = −1.151), age (t = −0.473), or BMI (t = −1.093). These findings ensured that the groups were comparable prior to the intervention. Table 1 displays the participants’ baseline demographic characteristics, and Figure 1 illustrates the flow of participant allocation.

### 2.2. Evaluation and Data Processing

In this study, only the affected limb—identified as having more severe symptoms based on radiological and clinical diagnoses in medical institutions—was measured and included in the analysis. The primary outcome measure was the YBT, which was used to assess dynamic balance performance. The secondary outcome measures were the COP velocity during the YBT and the VAS score for pain intensity.

#### 2.2.1. Y-Balance Test

Dynamic balance performance was assessed using the YBT. Participants performed the test barefoot while standing on one leg and reaching in three specified directions: ANT, PM, and PL.

The reaches were performed in a fixed sequence of ANT, PM, PL, five trials were conducted in each direction, and the average of three valid trials was used for data analysis. A trial was deemed invalid if the participant lost balance, touched the floor with the non–stance foot, or moved the stance leg during the reach. In such cases, the trial was repeated under the same conditions. To minimize fatigue, a 30 s rest interval was provided between directions.

**Figure 2:**
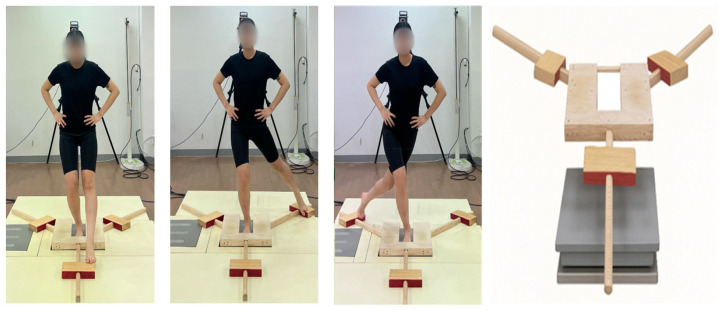
Combined assessment of the YBT and COP.

In this study, the composite reach distance was derived by summing the ANT, PM, and PL reach distances, dividing the total by three times the leg length, and converting the result to a percentage to account for individual limb length differences (Figure 2) [19].

#### 2.2.2. Center of Pressure Analysis

In this study, the YBT apparatus was placed on a force platform (AMTI OR6, Watertown, MA, USA) to simultaneously collect COP displacement data. COP data were recorded at a sampling frequency of 100 Hz, and a fourth-order Butterworth low-pass filter with a cutoff frequency of 6 Hz was applied to remove signal noise. In addition, to account for differences in movement speed among participants, COP velocity was normalized to each individual’s reach speed during the YBT. The analyzed variables included the mean COP velocity in the anterior–posterior (AP) and medial–lateral (ML) directions.

#### 2.2.3. Visual Analogue Scale

Pain intensity was assessed using the VAS. The VAS consists of a 10 cm horizontal line, anchored with “no pain” at the left end (0) and “worst imaginable pain” at the right end (10). Participants were instructed to mark the point on the line that best represented their current level of pain, and the recorded values were used for statistical analysis.

### 2.3. Exercise Intervention

The exercise in this study was performed by walking back and forth along a 10 m straight course on level ground, which was designed with reference to the distance-based walking assessment protocol used in the Health ABC Long Distance Corridor Walk study by Simonsick et al. [30], where participants repeatedly traversed a 20 m corridor. The walking exercise program consisted of a 10 min warm-up, 30 min main exercise, and 10 min cool-down, conducted twice per week for eight weeks (a total of 16 sessions). The exercise intensity was set at a Rating of Perceived Exertion (RPE) of 9–11 (light intensity) with a 3 kg load during weeks 0–4 and was progressively increased to an RPE of 12–13 (moderate intensity) with a 4 kg load during weeks 5–8 [31].

Accordingly, to gradually increase the total exercise volume, the number of laps was progressively expanded—five laps during weeks 1–2, six during weeks 3–4, seven during weeks 5–6, and eight during weeks 7–8.

**Figure 3:**
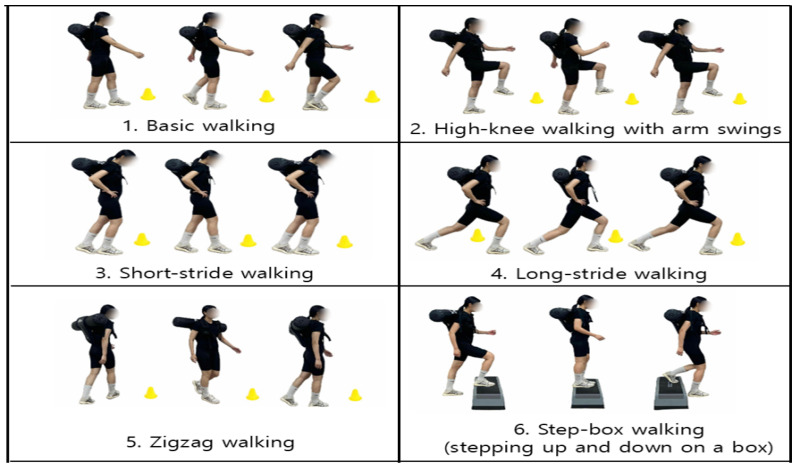
Walking exercise program performed for 8 weeks.

The detailed contents of the program are presented in Table 2 and Figure 3.

Participants attended an average of 15.5 out of 16 sessions, resulting in a compliance rate of approximately 96.6%. All sessions were conducted under the supervision of an experienced instructor, and no injuries or adverse events occurred during the intervention period.

The experimental group wore an Aqua Vest (Smartfits, Busan, Republic of Korea) providing a water inertial load [27]. The device used in this study is commercially manufactured sports equipment, not classified as a medical device, and not a prototype developed for research purposes. It is partially filled with water to generate inertial perturbations during movement. The control group wore a fixed weighted vest.

**Figure 4:**
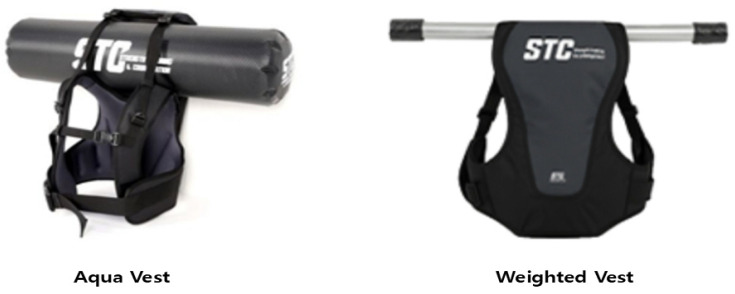
Differences in the vests used between the intervention groups.

Both groups performed the same exercise movements, order, and time structure; the only difference was the type of load (inertial vs. fixed), as shown in Figure 4.

### 2.4. Statistical Analysis

All statistical analyses were performed using SPSS Statistics 25.0 (IBM Corp., Armonk, NY, USA). Descriptive statistics were calculated as means ± standard deviations, and baseline homogeneity between groups was verified prior to the intervention.

Analysis of covariance (ANCOVA) was used instead of repeated-measures analysis of variance to control for baseline differences by including pre-test values as covariates and to reduce residual variance, thereby improving the precision of between-group comparisons. Before performing ANCOVA, the assumption of homogeneity of regression slopes was tested and confirmed.

Because the assumption of normality was not met, a nonparametric bootstrap resampling procedure (5000 resamples) was applied, and bias-corrected and accelerated (BCa) 95% confidence intervals were reported to account for skewness and bias in the sampling distribution. Within-group (pre–mid–post) changes were analyzed using the Wilcoxon signed-rank test. The level of statistical significance was set at *p* < 0.05.

Effect sizes for between-group comparisons (ANCOVA) were expressed as partial eta squared (*ηp*^2^), and effect sizes for within-group comparisons (Wilcoxon test) were expressed as r values. Partial eta squared (*ηp*^2^) values were interpreted as small (0.01), medium (0.06), and large (0.14), while r values were interpreted as small (0.10), medium (0.30), and large (0.50) [32].

## 3. Results

The results of this study for the YBT, COP, and VAS are presented in Table 3, Table 4 and Table 5, respectively.

### 3.1. Change in YBT

According to the ANCOVA results, in the mid-intervention phase (4 weeks), the Aqua Vest group showed a value of 88.65% (95% CI [85.87, 91.43]), while the control group showed 84.29% (95% CI [81.29, 87.28]), indicating a significant difference between the two groups (*p* = 0.041, *ηp*^2^ = 0.16).

At the post-intervention phase (8 weeks), the Aqua Vest group demonstrated 92.50% (95% CI [87.64, 97.36]), and the control group 82.89% (95% CI [77.65, 88.13]), with a significant difference again observed between the groups (*p* = 0.012, *ηp*^2^ = 0.23).

In the within-group comparison, the experimental group showed significant improvements in all intervals—pre- to mid-intervention (*p* < 0.001, *r* = 0.88), mid- to post-intervention (*p* = 0.036, *r* = 0.54), and pre- to post-intervention (*p* = 0.001, *r* = 0.88).

In contrast, the control group exhibited significant improvements in the pre- to mid-intervention (0–4 weeks; *p* = 0.001, *r* = 0.88) and pre- to post-intervention (0–8 weeks; *p* = 0.006, *r* = 0.77) intervals, whereas no significant change was observed between mid- and post-intervention (4–8 weeks; *p* = 0.972, *r* = 0.01).

### 3.2. Change in COP

According to the ANCOVA results, in the ANT direction, at the mid-intervention phase (4 weeks), the Aqua Vest group showed values of AP: 0.24 (95% CI [0.19–0.28], *ηp*^2^ = 0.09) and ML: 0.16 (95% CI [0.12–0.20], *ηp*^2^ = 0.11), while the control group showed AP: 0.29 (95% CI [0.24–0.34], *ηp*^2^ = 0.09) and ML: 0.21 (95% CI [0.17–0.26], *ηp*^2^ = 0.11).

At the post-intervention phase (8 weeks), the Aqua Vest group demonstrated AP: 0.20 (95% CI [0.17–0.24], *ηp*^2^ = 0.23) and ML: 0.15 (95% CI [0.12–0.18], *ηp*^2^ = 0.19), whereas the control group demonstrated AP: 0.27 (95% CI [0.23–0.31], *ηp*^2^ = 0.23) and ML: 0.20 (95% CI [0.17–0.23], *ηp*^2^ = 0.19).

Both groups showed a decreasing trend in AP and ML values over time, indicating reduced sway magnitude in the ANT direction.

### 3.3. Change in VAS

According to the ANCOVA results, at the mid-intervention phase (4 weeks), the Aqua Vest group showed a mean value of 2.01 (95% CI [1.39–2.62], *ηp*^2^ = 0.10), while the control group showed 2.76 (95% CI [2.10–3.43], *ηp*^2^ = 0.10).

The post-intervention phase (8 weeks), the Aqua Vest group demonstrated 1.77 (95% CI [1.09–2.45], *ηp*^2^ = 0.01), and the control group demonstrated 1.96 (95% CI [1.23–2.70], *ηp*^2^ = 0.01).

## 4. Discussion

This study aimed to investigate the effects of walking exercise using a water inertial load on dynamic balance ability and pain in women aged 65 years and older with KOA. As a result, the Aqua Vest group showed a significant improvement in YBT reach distance after eight weeks of intervention, indicating enhanced performance in the primary indicator of dynamic balance. For the secondary indicator, COP velocity showed a group difference in the ANT direction. In contrast, the PM and PL directions and pain (VAS) did not show statistically significant between-group differences; however, a decreasing trend with moderate to large effect sizes was observed, suggesting that walking exercise with water inertia load may have had positive effects on balance control and pain reduction.

The YBT is a representative indicator of dynamic balance that comprehensively reflects lower-limb proprioception, muscle strength, and neuromuscular control ability. It assesses an individual’s capacity to maintain a stable center of mass while reaching in three directions—anterior, posteromedial, and posterolateral—under a single-leg support condition [19,20]. In adults aged 65 years and older, the YBT has been presented as a useful tool for predicting fall risk and evaluating functional mobility, as declines in lower-limb strength and sensorimotor integration directly affect balance performance [21,22]. In the walking exercise applied in this study, the Aqua Vest generated irregular inertial resistance due to the internal movement of water, requiring continuous postural adjustments and neuromuscular responses throughout the exercise.

This likely led to improvements in balance control during YBT performance. These findings can be explained by the kinematic similarities between walking movements and the directional characteristics of the YBT. The ANT direction involves supporting body weight on one leg while reaching the opposite leg forward, which is closely related to forward propulsion in gait. Nelson et al. [33] reported that knee flexion and contralateral trunk rotation are the main kinematic factors during ANT reaching, and that knee extension and hip abduction moments are key kinetic predictors. This sagittal plane–dominant movement pattern resembles the propulsion phase of gait, during which the stance leg pushes the body forward. In addition, the PM and PL directions reflect medial–lateral stability of the stance leg during single-leg support and particularly require coordinated control between the hip abductors and ankle joints during weight transfer. These results are consistent with the findings of Olszewski et al. [34], who reported that YBT performance in the PM and PL directions can be significantly predicted by hip abductor strength and functional coordination of the ankle joint.

Therefore, the hip abductors are interpreted to have contributed to improved performance in the PM and PL directions by maintaining pelvic stability during single-leg support and assisting center-of-mass control in coordination with the ankle joint. The walking exercise in this study consisted of repetitive forward movements, and when wearing the aqua vest, the inertial load of the water generated resistance from multiple directions during motion, requiring continuous postural control.

Repeated exposure to such a resistive and unstable environment likely led to improvements in balance performance across all three YBT directions (ANT, PM, and PL). These findings are consistent with those of Gerards et al. [35] and Pai et al. [36], who reported that training under unpredictable perturbation conditions enhances both reactive balance control and anticipatory postural adjustment abilities. This suggests that similar balance control mechanisms operated in the perturbation-based exercise environment applied in this study.

Accordingly, the walking exercise with water inertia load induced unpredictable perturbations with each step, requiring continuous adjustments of the body’s center of mass. Repeated exposure to this unstable environment is meaningful in that it trains the body to anticipate and adapt to postural changes [37], thereby enhancing anticipatory postural control. In contrast, the control group’s fixed weighted vest provided a constant load with insufficient perturbation stimuli, which likely limited multidirectional sensory input and adaptive responses.

The secondary outcome analysis of the COP revealed a significant improvement in the ANT direction during YBT performance. The walking exercise in this study consisted of repetitive forward movements on a flat surface and involved an environment that required continuous adjustment to unpredictable perturbations caused by the inertial load of water. Such structural characteristics of the exercise are believed to have repeatedly stimulated postural control and balance maintenance in the anterior direction. Neptune et al. [15] reported that the ankle plantar flexors play a crucial role in maintaining dynamic balance in both the sagittal and frontal planes during gait. Therefore, the walking exercise with water inertia load in this study likely promoted improvement in anterior balance ability by repeatedly activating these movement patterns.

In contrast, no group differences were found in the PM and PL directions. This may be due to the relatively short reach distance of the YBT task, which might not have sufficiently reflected changes in postural control ability through COP variation during task performance. Nevertheless, this study complemented the limitations of conventional balance assessments that relied on a single indicator by additionally analyzing COP variations along with improvements in YBT reach distance.

The COP represents the point of application of ground reaction forces between the foot and the surface, serving as a quantitative measure of postural stability and balance control [38].

In particular, the temporal rate of change (velocity) of the COP has been proposed as a sensitive indicator of balance control in dynamic situations [24]. In this study, COP analysis provided meaningful insight by numerically reflecting changes in real-time weight-shift control that could not be identified by reach distance alone.

In this study, the integrated use of YBT and COP allowed for a more systematic understanding of the biomechanical characteristics of dynamic balance ability. The water inertia load and unstable perturbation environment may have stimulated proprioceptive input from the ankle, knee, and hip joints, inducing sensorimotor readjustment. The improvement in neuromuscular coordination was reflected in the decreasing trend of COP velocity, suggesting a potential enhancement in weight-shifting control through the integration of feedforward and feedback mechanisms.

The secondary outcome of pain showed no significant differences between the two groups; however, both demonstrated a decreasing trend. This may be because both the aqua-vest and weighted-vest exercises promoted regular activation of the lower-limb muscles and improved joint mobility, thereby contributing to reduced pain sensitivity [39].

In other words, rather than the difference in load type, the physiological effects of repetitive lower-limb exercise itself appear to have had a greater impact on pain reduction. Wang [40] reported that even mild KOA can induce pain responses due to intra-articular structural damage, and that pain intensity is closely associated with joint stability. From this perspective, both types of load-bearing walking exercises in this study may have alleviated pain by enhancing joint stability through activation of periarticular muscles and stimulation of proprioceptive feedback. In particular, walking with water inertia load, due to its unstable resistance characteristics, may have required more diverse neuromuscular control strategies, thereby providing additional benefits in pain reduction and balance improvement.

This finding is consistent with Lin [13], who reported the pain-relieving effects of proprioceptive exercises. Meanwhile, walking exercise is known to be one of the most common physical activities among adults aged 65 years and older [25], although Wallis et al. [26] pointed out that walking alone has limitations in improving pain in patients with KOA.

Therefore, regular walking exercises utilizing both water inertia and weighted loads may serve as a practical approach to improving pain control and balance ability in women aged 65 years and older.

The limitations of this study are as follows.

1. The intervention period was limited to eight weeks, which may have been insufficient to fully capture long-term functional changes.
2. Exercise intensity was determined based on the subjective RPE, and objective physiological indicators were not incorporated.
3. Because pain assessment was limited to a subjective measure (VAS), future studies should include objective physiological measurement methods such as electromyography (EMG) analysis.
4. This study included only women aged 65 years and older as participants; therefore, generalization to men may be limited due to physiological differences between sexes.

This study demonstrated that walking exercise using water inertial load can activate neuromuscular control strategies that are difficult to elicit through conventional walking alone, and may serve as a practical intervention capable of simultaneously improving pain and balance ability. However, the possibility that pain may naturally decrease over time regardless of the intervention cannot be fully excluded, and the relatively short intervention period of eight weeks may have been insufficient to capture long-term functional or structural changes.

From a clinical perspective, this intervention may be particularly suitable for older adults with mild to moderate KOA who are able to ambulate independently and tolerate low to moderate exercise intensity. However, because participants with relatively low baseline pain levels were included in this study, caution is warranted when applying this intervention to individuals with severe pain or advanced disease. In addition, patients with advanced KOA (Kellgren–Lawrence grade 4) were excluded; therefore, generalization of the findings to more severe cases should be made with discretion.

Future studies should include longer intervention periods and employ objective physiological indicators—such as muscle activation, muscle fatigue, and joint loading—to more comprehensively verify pain modulation effects. Moreover, further investigation is needed to evaluate the safety and effectiveness of this intervention in patients with severe symptoms and to develop individualized intervention strategies based on pain severity and functional capacity.

In particular, because research on perturbation-based training using water inertial load in patients with KOA remains extremely limited, future studies should expand intervention duration and design programs incorporating multidirectional water inertial loads to more clearly elucidate the underlying mechanisms and effects.

Such an approach is expected to provide practical evidence for developing rehabilitation programs for older adults who require improvements in both dynamic stability and pain management.

## 5. Conclusions

The present study investigated the effects of walking exercise with water inertial load on dynamic balance ability and pain in women aged 65 years and older with KOA. After an 8-week intervention, the experimental group showed significant improvements in all three directions of the YBT, the primary outcome measure, indicating that the inertial load of water may contribute to enhanced dynamic balance capacity. This suggests that the nonlinear inertial load of water may be associated not only with muscle strengthening effects but also with improvements in neuromuscular control and sensory–motor integration. In addition, both groups demonstrated decreasing trends in the secondary outcome measures of COP and VAS. These findings indicate that walking exercise using water inertial load can serve as a supportive or alternative intervention to traditional walking exercise, contributing to the recovery of balance function and enhancement of functional stability.

## Figures and Tables

**Table 1:** General Characteristics of the Participants (*n* = 28).

	AG (*n* = 15)	CG (*n* = 13)	*p*
Age (years)	70.47 ± 1.77	70.77 ± 1.59	0.640
Weight (kg)	55.73 ± 5.84	59.01 ± 8.37	0.914
Height (cm)	154.39 ± 4.16	154.55 ± 3.91	0.273
BMI (kg/m^2^)	22.59 ± 2.67	23.83 ± 3.32	0.293

Data are presented as mean ± standard deviation. No significant differences were found between groups (*p* > 0.05). AG = Aqua Vest group and CG = Control (Weighted Vest) group.

**Table 2:** Walking Exercise Program.

Training	0~4 Weeks/Program	5~8 Weeks/Program	Time
Intensity	9~11 RPE With Weight (3 kg)	12~13 RPE With weight (4 kg)	
Laps	Weeks 1–2: 5 laps Weeks 3–4: 6 laps	Weeks 5–6: 7 laps Weeks 7–8: 8 laps	
Warm up	1. Ankle and knee rotations 2. Hip joint rotations 3. Marching in place with arm swings 4. Trunk rotations with shoulder rolls	1. Ankle and knee rotations 2. Hip joint rotations 3. Marching in place with arm swings 4. Trunk rotations with shoulder rolls	10 min
Main	1. Basic walking 2. High-knee walking with arm swings 3. Short-stride walking 4. Long-stride walking 5. Zigzag walking 6. Step-box walking (stepping up and down on a box)	1. Basic walking 2. High-knee walking with arm swings 3. Short-stride walking 4. Long-stride walking 5. Zigzag walking 6. Step-box walking (stepping up and down on a box)	30 min
Cool down	1. Calf stretching 2. Hamstring stretching 3. Quadriceps stretching 4. Ankle pumping and rotation	1. Calf stretching 2. Hamstring stretching 3. Quadriceps stretching 4. Ankle pumping and rotation	10 min

**Table 3:** ANCOVA and Wilcoxon test results for Y-Balance Test composite scores between and within groups.

Variables Time	AG Adjusted Mean (CI 95%)	CG Adjusted Mean (CI 95%)	*p*	*ηp* ^2^	Wilcoxon (AG) *p*	*r*	Wilcoxon (CG) *p*	*r*
YBT Mid	88.65 (85.87–91.43)	84.29 (81.29–87.28)	0.041	0.16	0–4 weeks 0.001	0.88	0–4 weeks 0.001	0.88
YBT Post	92.50 (87.64–97.36)	82.89 (77.65–88.13)	0.012	0.23	4–8 weeks 0.036	0.54	4–8 weeks 0.972	0.01
					0–8 weeks 0.001	0.88	0–8 weeks 0.006	0.77

Note: Values are adjusted means (95% CI). YBT values are normalized to leg length and expressed as percentages. ANCOVA was used to compare between-group differences, and Wilcoxon signed-rank tests were used for within-group comparisons across time intervals. AG, Aqua Vest group; CG, Control (Weighted Vest) group; *ηp*^2^, partial eta squared; *r*, effect size. For interpretation, *ηp*^2^ values of 0.01, 0.06, and 0.14 indicate small, medium, and large effects, respectively, and *r* values of 0.1, 0.3, and 0.5 indicate small, medium, and large effects, respectively.

**Table 4:** ANCOVA results for COP velocity (anterior–posterior and mediolateral) during YBT tasks.

	Variables	Time	AG Adjusted Mean (CI 95%)	CG Adjusted Mean (CI 95%)	*p*	*ηp* ^2^
ANT	AP Velocity	Mid	0.24 (0.19–0.28)	0.29 (0.24–0.34)	0.120	0.09
	(m/s)	Post	0.20 (0.17–0.24)	0.27 (0.23–0.31)	0.012	0.23
	ML Velocity	Mid	0.16 (0.12–0.20)	0.21 (0.17–0.26)	0.096	0.11
	(m/s)	Post	0.15 (0.12–0.18)	0.20 (0.17–0.23)	0.022	0.19
PM	AP Velocity	Mid	0.21 (0.18–0.24)	0.24 (0.20–0.27)	0.162	0.08
	(m/s)	Post	0.22 (0.18–0.25)	0.23 (0.20–0.27)	0.437	0.02
	ML Velocity	Mid	0.12 (0.09–0.14)	0.15 (0.12–0.18)	0.098	0.11
	(m/s)	Post	0.12 (0.10–0.14)	0.15 (0.13–0.17)	0.096	0.11
PL	AP Velocity	Mid	0.24 (0.20–0.28)	0.27 (0.23–0.31)	0.366	0.03
	(m/s)	Post	0.25 (0.20–0.29)	0.26 (0.22–0.31)	0.603	0.01
	ML Velocity	Mid	0.11 (0.08–0.13)	0.14 (0.11–0.16)	0.065	0.13
	(m/s)	Post	0.12 (0.10–0.15)	0.13 (0.10–0.15)	0.693	0.01

Note: Values are adjusted means (95% CI). COP variables represent anterior–posterior (AP) and mediolateral (ML) velocities (m/s) measured during the Y-Balance Test (YBT) tasks performed in the anterior (ANT), posteromedial (PM), and posterolateral (PL) directions. ANCOVA was used to compare between-group differences at mid- and post-intervention time points. AG, Aqua Vest group; CG, Control (Weighted Vest) group; *ηp*^2^, partial eta squared. For interpretation, *ηp*^2^ values of 0.01, 0.06, and 0.14 indicate small, medium, and large effects, respectively.

**Table 5:** ANCOVA results for VAS pain scores between groups.

Variables Time	AG Adjusted Mean (CI 95%)	CG Adjusted Mean (CI 95%)	*p*	*ηp* ^2^
VAS Mid	2.01 (1.39–2.62)	2.76 (2.10–3.43)	0.115	0.10
VAS Post	1.77 (1.09–2.45)	1.96 (1.23–2.70)	0.702	0.01

Note: Values are adjusted means (95% CI). VAS scores represent perceived knee pain intensity measured during daily activities. ANCOVA was used to compare between-group differences at mid- and post-intervention time points. AG, Aqua Vest group; CG, Control (Weighted Vest) group; *ηp*^2^, partial eta squared. For interpretation, *ηp*^2^ values of 0.01, 0.06, and 0.14 indicate small, medium, and large effects, respectively.

## Data Availability

The data presented in this study are available on request from the corresponding author. The data are not publicly available due to privacy and ethical restrictions.
